# A Quick Guide to Genomics and Bioinformatics Training for Clinical and Public Audiences

**DOI:** 10.1371/journal.pcbi.1003510

**Published:** 2014-04-10

**Authors:** Michelle D. Brazas, Fran Lewitter, Maria Victoria Schneider, Celia W. G. van Gelder, Patricia M. Palagi

**Affiliations:** 1 Ontario Institute for Cancer Research, Toronto, Canada; 2 Whitehead Institute for Biomedical Research, Massachusetts Institute of Technology, Cambridge, United States of America; 3 The Genome Analysis Centre, Norwich, United Kingdom; 4 Netherlands Bioinformatics Centre and Department of Bioinformatics, Radboud Medical Center, Nijmegen, The Netherlands; 5 SIB Swiss Institute of Bioinformatics, Geneva, Switzerland; Philadelphia, United States of America

## Introduction

Traditionally, bioinformatics tools and training programs have focused on life science audiences. Though heterogeneous, their needs are at least fairly well understood. Driven by the impact of technology in diverse areas, bioinformatics is becoming increasingly interdisciplinary, and, in parallel, so too are the audiences seeking bioinformatics training. Audiences as disparate as physicians and lawyers, industry, and even the general public, previously without real need of bioinformatics skills or awareness, are now pursuing an understanding of and skill sets in bioinformatics.

These audiences represent a new and exciting challenge for bioinformatics training programs. A recent workshop at ISMB/ECCB (Intelligent Systems for Molecular Biology/European Conference on Computational Biology) 2013, “Workshop on Education in Bioinformatics 2013” (WEB2013), discussed opportunities and bioinformatics training strategies for emerging clinical and public audiences [1]. The aim of this Quick Guide is to share our guidelines for core bioinformatics skills and training requirements with bioinformatics educators and trainers who are already involved in or are thinking about developing and delivering bioinformatics programs to these audiences.

## The Healthcare Case: Scientific Advances Create a Bioinformatics Training Need

The contributions of genomics and bioinformatics to our understanding of biology and its role in disease have expanded significantly since the publication of the reference human genome [2], [3]. Next-generation sequencing and other high-throughput genomics technologies, including genome-wide association studies and RNA sequencing, have become ubiquitous in biomedical research [4], [5]. The clinical application and healthcare impact of these genomic technologies extends beyond Mendelian disorders [6] to common complex diseases, such as cancer [7] and autism [8], as well as to biomarkers for adverse events [9], [10], vaccine design [11], and the dosing [12] of therapies. Indeed, whole-genome sequencing and exome sequencing are emerging as valuable tools in personalized medicine or precision therapy [13], [14].

However, as recently noted [15], profound improvements in the effectiveness of genomics on healthcare cannot be realistically achieved without new policies, practices, and developments. Key among the gaps highlighted as fundamental to the advancement of genomics in the clinical setting were bioinformatics, computational biology, education, and training [15].

For the Global Organization for Bioinformatics Learning, Education, and Training (GOBLET) and the International Society for Computational Biology (ISCB), these gaps represent training opportunities for clinical and public audiences. WEB2013 included speakers well versed in providing bioinformatics training to these audiences: Dr. Russ Altman (Stanford University) and Dr. Donna Slonim (Tufts University) addressed the bioinformatics training needs of the clinical audience; and Hienke Sminia (Netherlands Bioinformatics Centre) and Dr. Winston Hide (Harvard School of Public Health) presented their perspectives on the bioinformatics training needs of the public. This initial set of guidelines is intended to stimulate further conversation on the core competencies and training opportunities for these key audiences. As it applies to healthcare, these efforts would help genomic medicine become an integral and effective part of medical care, mutually understood by healthcare providers and public recipients alike.

## Needs from the Clinical Audience

As recently noted, “intricate analyses of a patient's genomic data are destined to become an integral part of routine medical practice” [14]. Despite these projections and early demonstrations of clinical utility, broad translation of genomic medicine into the clinical setting has not yet been seen. One challenge to moving from base pairs to bedside [15] is the education of healthcare professionals: healthcare practitioners require the ability to interpret genomic data and make evidence-based decisions from this data. Healthcare professionals recognize their limitations in evaluating genomic data and readily seek training opportunities, not to become bioinformaticians as through biomedical informatics programs, but to become knowledgeable users who can understand the output from bioinformatic analyses of genomic data and competently make data-driven medical decisions.

**Figure 1:**
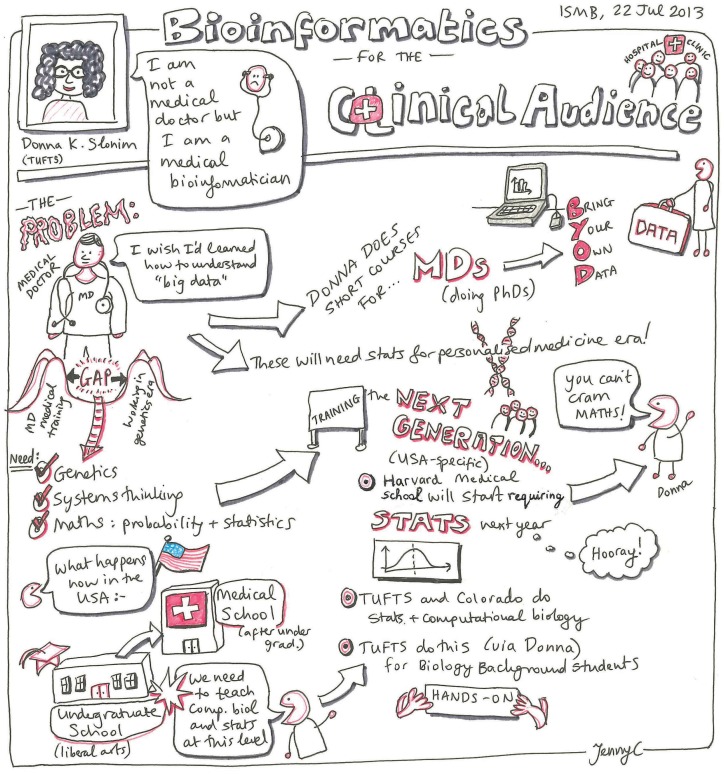
Genomics and bioinformatics for the clinical audience. A sketch of Donna Slonim's presentation on the genomics and bioinformatics needs for the clinical audience from the ISMB/ECCB 2013 conference. Image attributed to and used with the permission of Jennifer A. Cham [32].

During WEB2013, Altman and Slonim explored the core competencies in bioinformatics education and training required to achieve such fluency (Figure 1). Altman pointed out that the introduction of genomic-based assays into the clinic has necessitated changes in the electronic medical record (EMR), from the basic EMR, comprising just a patient's family history, medications, and procedures, to the enhanced EMR, which may now also include detailed genomic and molecular information. This shift will continue as whole-genome and exome sequencing of patients becomes common practice in the clinical setting. As a result of these changes in medical record keeping, Altman discussed the need for clinical professionals to gain an appreciation of and level of comfort in working with various scales of “big data.” An appreciation of big data (Table 1) would allow physicians to not only understand a patient's genomic data and its relationship to his/her health but to also make inquiries and seek answers using larger cohort data accessible through EMRs, thereby opening up the potential for broader medical breakthroughs [16]. Altman noted that “translational or clinical bioinformatics” programs (of which there are many [17]) are of great interest to medical students and funding bodies alike. Some of these funding bodies even set participation requirements for students to retain funding. For example, some NIH (National Institutes of Health) training grant programs allocate slots specifically to students with clinical training.

**Table 1:** Genomics and bioinformatics training needs for the next-generation clinical audience with a quick guide to training approaches.

Bioinformatics Training Needs	Quick Guide to Clinician Training
Statistics foundation	• Add statistics course requirements pre-medical school
	• Offer statistical primers in continuing education training
	• Develop competency in experimental design, hypothesis testing, and statistical significance
Genetics and genomics foundation	• Expand requirements for genetics and genomics coursework in medical school
	• Offer genetics and genomics primers in continuing education training
	• Maintain currency of genetics and genomics materials as these are rapidly changing fields
	• Use genomics-based case examples to illustrate concepts in genetics coursework
Programming basics	• Offer UNIX, Python, and R skills in short courses post-medical school
	• Develop basic programming skills for file manipulation and automating repetitive tasks and understand the difference between structured and unstructured data
Big Data appreciation	• Include short projects combining all of the above skills applied to a cohort of EMR data or clinical case examples in medical school projects and in continuing education training

Slonim furthered the idea of clinicians being able to work with and understand big “medical” data. She argued that to better prepare medical students for translational informatics and data-driven medicine, there is a need for better training in the fundamentals of statistics and genetics (Table 1). Others at Yale and Stanford have made similar statements, focusing on the need to develop clinicians who are adaptive, creative thinkers prepared to integrate new scientific advances made throughout their careers [18]. Dienstag from Harvard notes that if medical schools are to have the freedom to equip students for the practice of scientifically anchored medicine, then students should arrive with a higher level of scientific competency [19]. Slonim felt that setting different mathematical requirements for medical school admission and adjusting medical training to make use of relevant genomic-based case examples could then be followed up with undergraduate bioinformatics curricula such as those offered at Tufts and elsewhere (examples in Table 1 and Table S1) to achieve bioinformatically skilled physicians more familiar with systems-level thinking. She stressed that “mathematical maturity” can only be developed over time. As such, mathematic coursework is less suitable than genetics or systems biology to integration into the already-crowded medical curriculum or to short course training aimed at practicing clinicians. Developing the quantitative, mathematical skills needed for future medical careers is therefore best done over the course of undergraduate training.

Interestingly, a recent review of the Medical College Admission Test (MCAT) [20] drew similar conclusions. The review considered which molecular biology, genetics, and statistical topics were important for ensuring an entering student would have the ability to master the medical schools' current and future curriculum. Many of the topics flagged as critical for medical school entrance competency overlap with Altman and Slonim's views on training clinical professionals in genomics and bioinformatics [20]. A revised MCAT is expected in 2015, with recommended changes in competencies of future physicians having already been outlined [21]. Similar updates to training programs are being completed in other countries such as the United Kingdom, where the National Health Service has initiated a “Modernizing Scientific Careers” program (Table S1) in recognition that advances in scientific technology and changes to the delivery of healthcare scientific services have altered the boundaries between traditional healthcare and science workforce sectors [22].

## Needs from the Public Audience

**Figure 2:**
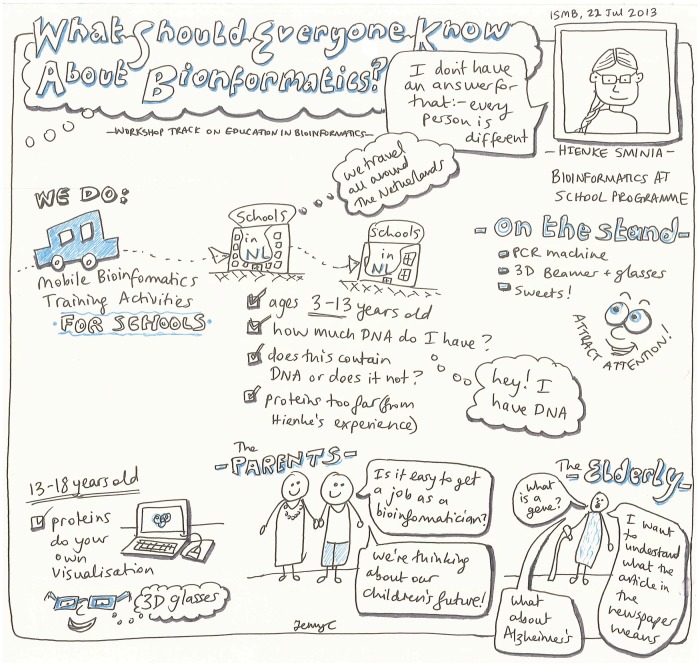
What should everyone know about genomics and bioinformatics? A sketch of Hienke Sminia's presentation on the genomics and bioinformatics needs of the public audience from the ISMB/ECCB 2013 conference. Image attributed to and used with the permission of Jennifer A. Cham [32].

Strengthening the public's scientific literacy, particularly in genomics and bioinformatics, is the reciprocal of training the clinical audience. Such literacy is important to achieve “an educated public who can understand the implications of genomics for their healthcare and evaluate the relevant public policy issues” [15]. Certainly, public hunger for learning about genomics (and indirectly bioinformatics) continues to grow with each media report on the progress of genomics in health and what such discoveries or information could mean on a personal level. Such was the case after Angelina Jolie published her experience with genetic screening for BRCA-1 gene mutations and subsequent decision to undergo a double mastectomy [23]. Targeting the public, however, presents its own challenges, particularly when the public often lacks an understanding of genetics, probability, and risk (e.g., the Jolie effect) and is constantly fed with misinformation from the media (e.g., skin-care advertisements that claim to repair your skin's DNA to reverse the effects of aging). At WEB2013, Sminia and Hide explored how to achieve better genomics and bioinformatics literacy through education and outreach with the public (Figure 2).

One long-term solution to improving public literacy is to bring genomics and bioinformatics into the formal high school classroom. Many initiatives around the world exist to educate high school pupils and their teachers [24]–[27].

Sminia drew upon her experiences of engaging with public audiences in genomics and bioinformatics activities in the Netherlands. Sminia pointed out that opportunities to engage with the public are often limited in time. Learning activities and the information they impart must therefore be brief, enticing, interactive, and engaging (Table 2). The public audience is broad and can be divided into distinct age groups, each with unique interests and different educational or awareness needs. Bioinformatics materials and activities developed for the public must therefore be made age appropriate (examples in Table 2 and Table S2).

**Table 2:** Genomics and bioinformatics awareness and educational needs for the public audience with a quick guide to training approaches.

Awareness and Educational Needs	Quick Guide to Public Education
Understandable explanations of molecular biology basics, genomics, and translational health informatics	• Simplify concepts
	• Avoid jargon
	• Present concepts in a clear, concise manner
	• Use information and activities appropriate to the engagement level of the age group
Open, accessible information	• Make materials open access
	• Post materials online
	• Use multiple social media resources
	• Use communication channels suitable for the audience's age group
Informative, engaging content	• Simplify concepts
	• Present single message activities
	• Attract audience attention with eye-catching displays
	• Use provocative questions to engage the audience
	• Relate concepts to current events in society or age group to provide context
	• Use interactive, hands-on activities to illustrate and demonstrate concepts
	• Develop age-appropriate content
Trusted information sources	• Educators such as GOBLET should constantly post or collect trusted materials addressing specific areas of public need
	• Educators should endeavour to build their trusted brand

Bioinformatics concepts particularly amenable to public outreach are those that focus on molecular biology and its impact on public or personal health, as well as those that address ethical consequences and policy considerations affecting daily life. Enticing the public with current questions from news items (e.g., Do I need to be screened for BRCA-1 mutations like Angelina Jolie?) or with common queries and concerns (e.g., How are new drugs designed? Is there a test to determine if I will get Alzheimer's? Who will pay for these new genomic tests?) is helpful in drawing in the public to engage further with genomics and bioinformatics content. Again, all learning and awareness content should be constructed with the audience's age and background knowledge in mind.

Hide [28] urged that the time is ripe for bioinformatics educators, trainers, and research scientists to take advantage of the public's interest in our field and become a trusted source for explanations and learning materials on genomics and bioinformatics. He pointed out that becoming such a source would require more effective interaction through all recognized channels of public communication and news digestion, especially online resources like social media, blogs, massive open online courses, and YouTube (Table 2). With so many channels of information available, the public also wants to know where to find reputable materials that others like and understand. The implications for bioinformatics educators and research scientists alike is to create accessible, accredited, and reusable materials that bring awareness and education to the public and that speak to the public audience's learning interests and needs (Table 2). This focus has been the impetus for GOBLET. For university-level audiences (and possibly other classroom-environment audiences as well), Hide noted that the traditional didactic learning model may very well flip such that the majority of genomics and bioinformatics learning takes place outside of the classroom through simplified online resources and activities, leaving the classroom to become dedicated to small-group learning interactions. It was also noted that the public would continue to turn to their physicians for guidance and education on the health implications of their precision genomics and genetic tests, reinforcing the need for genomics and bioinformatics training of this group of healthcare professionals. Since big biological data is also predicted to become big business [29], direct-to-consumer genomics services (e.g., 23andMe) are spending enormous resources on communicating complex scientific data to their web-savvy clients [30]. Ensuring all public members have access to similar information and learning materials will be prudent.

## Outlook

Providing public and clinical audiences with well-informed, understandable, and reputable information and educational materials on genomics and bioinformatics topics is an immediate necessity. As bioinformatics trainers and educators, it is our responsibility to ensure the growing educational (for the public) and training (for the clinician) needs of these audiences are met. The guidelines presented here should serve as an initial set of pragmatic steps for creating engaged and well-informed public and healthcare communities. Such skills and awareness will go a long way in ensuring base-pair technologies translate rapidly to bedside, and they will bring to public light the importance of our field in the larger research arena.

All presentations from WEB2013 areopenly accessible and can be found on the GOBLET training portal at mygoblet.org [31].

## Supporting Information

Table S1 **Examples of genomics and bioinformatics coursework and continuing education programs for future and practicing clinical audiences.**
(XLSX)

Table S2 **Examples of genomics and bioinformatics educational awareness and educational resources for the public audience.** Many other excellent examples exist in numerous languages.
(XLSX)
